# 1028. Performance and Patient Acceptability Evaluation of the Chembio DPP® HIV-Syphilis Assay in an Emergency Department

**DOI:** 10.1093/ofid/ofab466.1222

**Published:** 2021-12-04

**Authors:** Kendall N Maliszewski, Yu-Hsiang Hsieh, Deanna Myer, Danielle A Perez, Charlotte A Gaydos, Yukari C Manabe, Erin Ricketts, Richard E Rothman

**Affiliations:** 1 Johns Hopkins University, Baltimore, Maryland; 2 Johns Hopkins, Baltimore, MD; 3 Johns Hopkins University School of Medicine, Baltimore, MD

## Abstract

**Background:**

Emergency departments (EDs) serve as sentinel settings for diagnosing sexually transmitted infections (STIs), including HIV and syphilis. We aimed to assess performance and patient acceptability of a point-of-care (POC) test, the Chembio Dual Path Platform (DPP®) HIV-Syphilis Assay, in an urban ED in Baltimore.

**Methods:**

170 patients were enrolled via convenience sampling from Oct 2019 – March 2020 and Jan 2021 – June 2021. Patients eligible were < 70 yrs, men who have sex with men, pregnant without care, had STI concerns, or history of drug use. Subjects received standard of care (SOC) HIV and syphilis testing under institutional laboratory algorithms. Subjects were then tested with the finger-stick POC test and completed a survey, both before and after the POC test to assess subjects’ attitudes about the POC test.

**Results:**

Comparing the SOC and POC results, 165/170 (97.1%) were test concordant. 3 syphilis POC results were false negative, but reported successful treatment over 10 years prior to enrollment (treponemal antibody remains after treatment). 1 HIV result was false negative and 1 was false positive. Overall the sensitivity and specificity of the HIV POC test were 96.8% (95%CI: 83.3%, 99.9%) and 99.3% (95% CI: 96.1%, 100%), and for syphilis were 85.7% (95%Cl: 63.7%, 97.0%) or 100% (95%CI: 81.5%, 100%), if excluding 3 persons having been successfully treated, and 100% (95% CI: 97.6%, 100%) respectively.

The pre-test survey found 67% and 77% of participants were comfortable with a finger-stick test and agreed the POC test result would be as good as the SOC test result, which increased to 96% and 86% in the post-test, respectively, (p< 0.05). At post-test, 86% reported they would feel confident to perform this test at home and 81% would use it at least once per year if it were available. 97% reported they were more likely to seek treatment if receiving a positive result during their ED visit and 91% reported it would reduce their stress/anxiety if receiving a negative test result in the ED.

**Conclusion:**

Our findings demonstrated satisfactory performance and high patient acceptability of the Chembio DPP® HIV-Syphilis Assay. Given the test is FDA approved, implementation studies are needed to determine whether adoption of this POC test will benefit patients and be consistent with ED workflows.

**Disclosures:**

**Richard E. Rothman, PhD, MD**, **Chem bio** (Grant/Research Support)

